# First-Order Reversal Curves of Sets of Bistable Magnetostrictive Microwires

**DOI:** 10.3390/ma16062131

**Published:** 2023-03-07

**Authors:** Ana María Cabanas, Rafael Pérez del Real, David Laroze, Manuel Vázquez

**Affiliations:** 1 Departamento de Física, FACI Universidad de Tarapacá, Arica 1010069, Chile; 2 Instituto de Ciencia de Materiales, CSIC, 28049 Madrid, Spain; 3 Instituto de Alta Investigación, CEDENNA, Universidad de Tarapacá, Casilla 7D, Arica 1010069, Chile

**Keywords:** magnetic microwires, FORC analysis, magnetostatic interactions, magnetic bistability

## Abstract

Amorphous microwires have attracted substantial attention in the past decade because of their useful technological applications. Their bistable magnetic response is determined by positive or negative magnetostriction, respectively. First-order reversal curves (FORC) are a powerful tool for analyzing the magnetization reversal processes of many-body ferromagnetic systems that are essential for a deeper understanding of those applications. After theoretical considerations about magnetostatic interactions among microwires, this work introduces a systematic experimental study and analysis of the FORC diagrams for magnetostrictive microwires exhibiting an individually bistable hysteresis loop, from a single microwire to sets of an increasing number of coupled microwires, the latter considered as an intermediate case to the standard many-body problem. We performed the study for sets of quasi-identical and different hysteretic microwires where we obtained the coercivity $H_{c}$ and interaction $H_{u}$ fields. In the cases with relevant magnetostatic interactions, FORC analysis supplies deeper information than standard hysteresis loops since the intrinsic fluctuations of the switching field generate a complex response. For sets of microwires with very different coercivity, the coercivity distributions of the individual microwires characterize the FORC diagram.

## 1. Introduction

The overall interest in amorphous metallic microwires arises from their excellent behavior as soft magnetic materials [1,2]. The most relevant magnetic properties are determined by their cylindrical shape, together with the strong mechanical stresses generated during the quenching from the melt fabrication process [3,4,5]. The coupling between such mechanical stresses, σ, with the material magnetostriction, $\lambda_{s}$, results in a very significant stored magnetoelastic energy density,

(1) $$K_{me}=\frac{3}{2}\lambda_{s}\sigma$$

Thus, ferromagnetic amorphous microwires are typically classified according to their magnetostrictive character defined by the spin-orbit coupling of the corresponding alloy. Particularly, Fe-based (e.g., FeSiB) alloys present a positive and high magnetostriction constant $\lambda_{s}=3\times 10^{-5}$, and their magnetization easy axis is parallel to the microwire’s axis within a large central core. At remanence, the microwire is essentially magnetized following one of its axis directions according to the effective uniaxial anisotropy. Under a small antiparallel applied magnetic field, the magnetization reverses direction. This bistable magnetic behavior takes place by the nucleation and depinning of a single domain wall (DW) in that core which gives rise to the observed squared-shaped hysteresis loop [6].

While the magnetostriction constant is an intrinsic property of the alloy composition, the mechanical stresses are generated during the quenching and drawing fabrication. Particularly, the difference between the thermal expansion coefficients of glass coating and the metallic nucleus diameter directly influences the level of internal stresses in Equation (1). Hence, microwires with identical metallic nucleus compositions may exhibit distinct magnetic properties because of the difference in the magnetoelastic energy resulting from different metallic diameters or thicknesses of the glass coating [4,7]. This feature leads to a broad spectrum of magnetic behavior, which is noteworthy for developing a wide variety of applications. Due to that bistable behavior, microwires are employed as sensing elements in sensor devices [8,9] or embedded in polymeric matrices [10].

A remarkable interest in amorphous microwires has also been stimulated by the magnetic interactions that appear when placing several of them in proximity [11,12]. These interactions arise from the stray fields surrounding each microwire due to net magnetic charges accumulated mostly at the ends, particularly in the case of magnetostrictive microwires. The modeling of interactions in microwire arrays is often subjected to strong simplification, for example, using the dipole approximation where the axial field generated by a microwire is proportional to its magnetization [13,14,15] or using a one-dimensional modified Ising model [11,14]. There, each microwire is considered a magnetic dipole. However, the magnetostatic dipolar field cannot quantitatively describe the interactions depending on the space between them. Consequently, in order to understand the experimental results, reliable methods for the evaluation of interactions are required.

To characterize the technical magnetic intrinsic properties of a system, the major hysteresis loop (MHL) is commonly measured. In addition, an alternative method based on the measurement and analysis of multiple minor hysteresis curves, called first-order reversal curves (FORC), has been recently intensively used to study information about magnetic phases, interactions, and the processes taking place during the magnetization reversal of ferromagnetic systems and other reversible and quasi-reversible systems [16,17,18,19,20,21,22,23,24,25].

**Figure 1:**
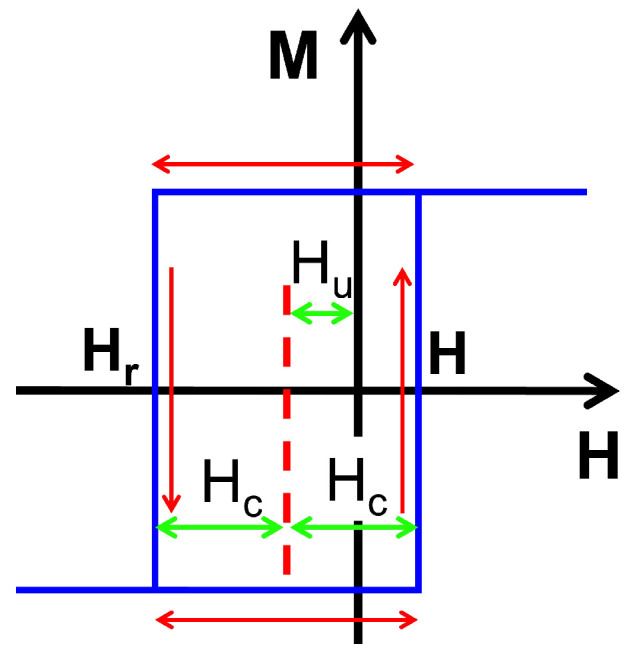
Scheme of mathematical hysteron corresponding to a bistable amorphous microwire. The green arrows denote the value of the coercive field $H_{c}$ and the bias field $H_{u}$. The red arrows correspond to the magnetization reversal.

The FORC diagram consists of a statistical distribution of square hysteresis loops completely confined inside the MHL. The hysteresis phenomenon is modeled by a set of operators, called hysterons (see Figure 1), which contain a physical meaning [26] or just a mathematical one [27] depending on the system. The hysterons are characterized by two parameters: an irreversible critical field, $H_{c}$, and an interaction field $H_{u}$. The magnetization switches up for $H=(H_{c}-H_{u})$ and switches down at $H_{r}=-\left(H_{c}+H_{u}\right)$. A FORC distribution ρ is interpreted as directly created by hysterons with dimensions and shapes that satisfy the physical set. Thus, to quantitatively analyze an experimental FORC diagram for amorphous bistable microwires and extract some physical parameters, we propose a squared irreversible hysteron, which provides a phenomenological description of the magnetic hysteresis in a single-domain particle of uniaxial anisotropy subjected to an external field. We consider that the (stable or metastable) equilibrium orientations of the spontaneous magnetization vector, M(r), under the effects of an applied field correspond to the minima of the free energy density.

Despite being a commonly used technique, a global consensus to unravel the information extracted from FORC diagrams is still lacking, notwithstanding the endeavor of several groups. In particular, there are some discrepancies in certain systems derived from the differential susceptibility curves [28] as soft ferromagnets or multiphase systems containing soft phases in which the interactions are comparable or larger than the switching fields [29].

This systematic experimental study introduces a broader discussion about the feasibility of FORC diagrams to analyze the interaction between sets of an increasing number of amorphous microwires with individually bistable behavior with the aim to link the FORC diagram results to the actual field of interactions between them and the intrinsic fluctuations of the switching field. For the sake of simplicity, we have chosen this particular system since the MHLs of these microwires are simple enough to provide a reliable comparison with the Preisach model [30,31]. Our results focus on the analysis of the experimental FORC technique and the features described by the FORC distribution.

## 2. Experimental Methods and Theoretical Calculations

### 2.1. Experimental Methods

Amorphous microwires were fabricated in our laboratory by quenching and drawing, also called the modified Taylor and Ulitovsky technique [3]. The microwires are composed of a metallic amorphous magnetic core with a diameter from 5 to 8 μm, inside a Pyrex glass-coating 5.5 to 7.5 μm thick that, apart from generating mechanical stresses to the core, provides electric and corrosion insulation. The ratio $\kappa =d_{m}/D$ takes a leading role in defining the magnetic behavior, where $d_{m}$ is the metallic core diameter and *D*, the total diameter of the microwire. In particular, we observed a larger coercivity in microwires with a thinner metallic nucleus and thicker glass coating and hence a smaller κ. The alloy composition selected for this investigation was Fe${}_{77.5}$B${}_{15}$Si${}_{7.5}$ with κ∈(0.25,0.4) showing large-positive ($\lambda_{s}=3.5\times 10^{-5}$) magnetostriction. The hysteresis loops measured for this selected composition give a bistable squared-shaped hysteresis loop, typical for magnetostrictive microwires.

The MHLs and the first-order reversal curves (FORC) measurements were taken in a vibrating sample magnetometer, VSM (ADE system, EV-7 KLA- Tencor) under a longitudinal applied magnetic field in which a maximum available field is 15 kOe. In FORC measurements, the maximum applied field (e.g., 5 Oe, 10 Oe, 15 Oe) is large enough to reach near magnetic saturation of the respective set of microwires. Pieces of microwires of 7 mm length were carefully cut with a scalpel. The chosen length, fitting in the magnetometer sample holder, is long enough to ensure the bistable magnetic behavior of the magnetostrictive microwires. Note that a minimum length of 3 to 4 mm is needed to observe a squared loop and avoid the collapse of closure domains appearing at the ends of the microwires, reducing their magnetostatic charges [5]. For the measurements of the MHLs of the different sets of microwires, the microwires were placed side by side in the same plane of the sample holder so that their metallic nucleus was separated by a distance equal to the thicknesses of the glassy coats.

### 2.2. Theoretical Approach

The usual approach for the description of the interaction between microwires is the dipolar interaction with some phenomenological modification [32]. Nevertheless, such a simple dipole approximation may lead to strong discrepancies when dealing with interacting microwires that are closely placed (e.g., the length of the microwires is much larger than the distance between them) [15]. We assume a simplified model in which L>>R ( *L* being the microwire length and *R* the radius). Hence, the discrete distribution of the microwire magnetic moments of the nucleus can be replaced by a continuous approach defined by M(r), in which the total magnetic moment within the element of volume δV centered at r is M(r)δV (where V is the volume of the metallic nucleus of the microwire). In addtion, $M\left(r\right)=M_{o}z$ defines the axial magnetization due to the shape anisotropy, where z is the direction along the microwire axis. With the aim of simplicity, we have considered a set of two microwires, whose magnetostatic interaction can be calculated from [33]:

(2) $$E_{int}=\mu_{o}\int M_{2}\left(r\right)\nabla U_{1}\left(r\right)dV$$

where $M_{2}\left(r\right)$ is the magnetization of the microwire 2, and $U_{1}\left(r\right)$ is the microwire 1 magnetostatic potential that can be written as:

(3) $$U=\frac{1}{4\pi }\left(-\int_{V}\frac{\nabla M(r^{\prime })}{|r-r^{\prime }|}d^{3}r^{\prime }+\int_{S}\frac{n^{\prime }M\left(r^{\prime }\right)}{|r-r^{\prime }|}ds\right)$$

In the case of a magnetically saturated microwire in the axial direction, the first term of the right-hand side of Equation (3) vanishes because the magnetization field is constant. Taking into account the symmetry of the problem and after straightforward calculations [34], the scalar potential can be approximated by:

(4) $$U=\frac{M_{o}R}{2}\int_{0}^{\infty }\frac{dk}{k}J_{o}\left(kr\right)J_{1}\left(kr\right)\left(e^{-k|\frac{L}{2}-z|}-e^{-k|-\frac{L}{2}-z|}\right),$$

where $J_{m}$ is a first-kind m order Bessel function. The magnetic field can be obtained from H(r)=−∇U(r), and the magnetostatic interaction energy, $E_{int}$, between the wires is given by:

(5) $$E_{int}=\int M_{2}H_{1}\left(r_{2}\right)dV_{2},$$

where the sub-indexes refer to microwires 1 and 2. The contribution of the magnetostatic energy is only due to a surface term since the magnetization field is assumed to be constant. Therefore, it is possible to use the integral expression of the scalar potential, Equation (3), and considering that in our case R/L≪1, we can approximate J[αx]≈αx/2, so that the energy between the two microwires becomes [35]:

(6) $$E_{int}=\frac{\mu_{o}m_{1}m_{2}}{2\pi L^{2}d}\left(1-\frac{1}{\sqrt{1+\frac{L^{2}}{d^{2}}}}\right),$$

where $m_{1}$ and $m_{2}$ are the magnetic moments of the microwires given by $m_{i}=M_{i}/V$, and *d* the interwire distance.

Let us first consider the simple case of two parallel microwires placed side by side in the presence of a positive and saturating magnetic field applied parallel to the axis of the microwires. The magnetization of both wires points in the same direction as the applied field. Afterward, under an antiparallel applied field, the magnetostatic interaction between them decreases the energy barrier, thereby increasing the probability of switching and favoring the magnetization reversal in one of the microwires [11]. However, such interaction enlarges the energy barrier for the reversal of the second microwire. Thus, the switching field, $H_{sw,i}$ at which the *i*-microwire reverses magnetization can be expressed as:

(7) $$H_{sw,i}=H_{0,i}+\epsilon H_{int,j},$$

where $H_{0,i}$ denotes the coercivity of the isolated individual *i*-microwire (i=1,2). We label $H_{sw,i}$ the switching field at which we observe the magnetization jumps of the MHLs, and $H_{c,i}$ to the coercive field described by the FORC diagram to remark the observed differences. Note that for a single microwire, its coercivity is $H_{c,1}=H_{sw,1}=H_{0,1}$. ϵ is an adjustable parameter that considers the non-uniform axial distribution of the magnetic charges along each microwire [36]. $H_{int,j}$ is the magnetostatic interaction field sensed by the *i*-microwire due to *j*-microwire (j=2,1), which can take positive or negative values. It can be written as a function of the magnetostatic interaction energy, $E_{int}$ as:

(8) $$H_{int,j}=\frac{\partial E_{int}}{\partial m_{i}}$$

In the case of sets of a larger number of microwires, the interaction field acting on each microwire becomes more complex to evaluate because of the discontinuities introduced by the magnetization reversal of individual microwires. A similar expression to Equation (7) is still valid, with $H_{int,j}$ being the addition of the interaction fields to the rest of the *j*-microwires. Therefore, in the case of i≥ 3, the value of the switching field $H_{sw,i}$ can be rewritten as:

(9) $$H_{sw,i}=H_{0,i}+\sum_{j}\epsilon H_{int,j}$$

### 2.3. FORC Diagrams

FORC diagrams are used to identify the Preisach distribution of a classical Preisach system based on the method proposed by Mayergoyz [30,31]. Although there are similarities between Preisach and FORC distributions, such as the sensitivity to magnetostatic interactions [19,21], FORC diagrams are less restrictive because of their entirely experimental procedure. Indeed, the FORC method does not assume that the distribution is symmetric about the $H_{c}$ axis, but the Preisach theory does. However, the vast majority of researchers have interpreted the FORC distribution as a lightly warped Preisach distribution. This concept has not been utterly proven, and their assumptions may be inaccurate, as discussed elsewhere [19,29].

To construct a first-order reversal curve, it is necessary to bring the magnetization, *M*, of the microwire in the ”up” position. Hence, each FORC is measured by first positively saturating the sample at a high positive saturation magnetic field $H_{max}$. The second step is to decrease the external applied field *H* until a point called reversal field $H_{r}$ and increase the applied field in ΔH steps up to a maximum field $H_{max}$ at which FORC curves are calculated once each microwire has reverted its magnetization. A FORC curve is obtained by measuring the magnetization as a minor loop from $H_{r}$ to the maximum field $H_{max}$ [37]. This process is repeated for equispaced reversal fields $H_{r}$ that increase in $\Delta H_{r}$ steps with $\left(-H_{max}<H_{r}<H_{max}\right)$. The magnetization as a function of $H_{r}$ and *H* is recorded at each step. In our measurements, the steps for the applied field are typically ΔH=0.05 Oe, and that of $\Delta H_{r}=0.1$ Oe, unless otherwise specified.

Lastly, a set of FORC inscribed as minor loops in the main MHL is recorded. The FORC distribution ρ is calculated by applying a second-order mixed derivative of $M(H_{r},H)$ regarding the applied field *H* and the reversal field $H_{r}$ [38]:

(10) $$\rho \left(H,H_{r}\right)=-\frac{1}{2}\frac{\delta M(H,H_{r})}{\delta H\delta H_{r}}\left(H>H_{r}\right),$$

which gives the variation of the differential susceptibility due to changes in the reversal field. The factor 1/2 is called for the identification of the FORC and Preisach distributions in the ideal case of systems satisfying the conditions from the classical Preisach model [30,31]. Commonly, to obtain a smooth FORC distribution, about 100 curves covering the hysteresis loop are necessary [16,21]. The FORC diagram is the contour plot representation of the distribution function ρ on the $H-H_{r}$ plane. Let us note that this derivative discards the purely reversible components of the magnetization in which ρ=0 [17,39]; therefore, the switching process will be described by ρ≠0 [40]. The usual representation of this distribution uses the switching fields $\left(H,H_{r}\right)$ as coordinated, named as “Preisach style”. However, many experimental works are described in “FORC style” using the interaction field axis:

(11) $$H_{u}=\frac{1}{2}\left(H+H_{r}\right)\;\;H_{c}=\frac{1}{2}\left(H-H_{r}\right)$$

This shift returns a rotation of the FORC diagram by $45^{\circ }$ and allows a more intuitive interpretation of the FORC distributions for any kind of system [19]. According to the classical Preisach model, see Figure 1, the hysteron is shifted along the field axis with the “bias field” $H_{u}$ reversing at $H_{r}=-\left(H_{c}+H_{u}\right)$ and switching back up at *H* $=(H_{c}-H_{u})$ as Figure 1 shows. This practice is supported because each point on the $\left(H,H_{r}\right)$ plane corresponds to a Preisach rectangular hysteron [30,31]. For all the diagrams in this work, we shall use the “FORC style” representation, where their accuracy is given by the magnetic field and reversal field steps, ΔH and $\Delta H_{r}$, respectively.

FORC diagrams are generally used in arrays of a large number of magnetic nano-systems (e.g., nanoparticles, nanowires) to unveil detailed information on the effects of the interaction field described by two characteristic regions or magnitudes [17,18,20]. For these systems, the first distribution is more prominent and corresponds to the interaction field distribution accounting for strong magnetostatic interactions. The second distribution, known as coercive field distribution, is related to non-interacting systems and has a large dispersion of coercive fields [19]. On the contrary, in our case, we are dealing with a discrete number of microwires that can be considered as interacting irreversible hysterons from the classical Preisach model [30,31]. Therefore, for the non-interacting case, we expect an extended coercive distribution along the $H_{c}$ axis at $H_{u}=0$, while for the interacting case, the distribution is located at $H_{u}\neq 0$ [41].

## 3. Experimental Results: Hysteresis Loops and FORC Analysis

### 3.1. Single Bistable Microwire

**Figure 2:**
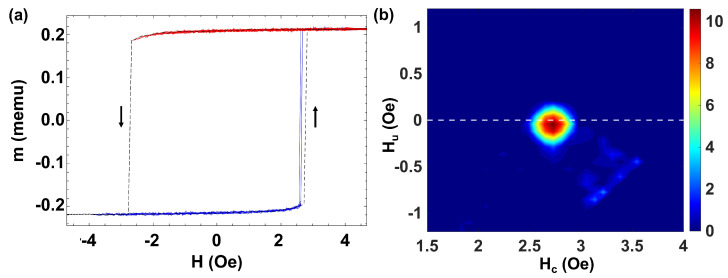
(**a**) MHL and FORC curves for a single Fe${}_{77.5}$B${}_{15}$Si${}_{7.5}$ bistable microwire with $H_{c}=2.73$ Oe when an axial magnetic field is applied. (**b**) Corresponding FORC diagram.

First, we consider the simplest case of a single individual bistable Fe${}_{77.5}$B${}_{15}$Si${}_{7.5}$ microwire. Figure 2a shows the experimental procedure to obtain the FORC curves. The black dashed line corresponds to the MHL with bistable character and giant Barkhausen jump at $H_{sw}=2.73$ Oe. The black arrows denote the direction in which the magnetization reversal proceeds. The blue lines correspond to the FORC curves in which the magnetization of the single microwire is reversed, while the red ones correspond to the magnetization values in which the applied reversal field is not strong enough to reverse the magnetization with a threshold value at $H_{r}=-2.68$ Oe.

Figure 2b shows the corresponding FORC diagram. The coercivity distribution is obtained from the peak of the FORC distribution located around $\left(H_{c},H_{u}\right)$ = (2.73,0) Oe [42,43]. Note that $H_{c}$ obtained from the FORC diagram takes the same value as the field for the magnetization switching or coercivity in the bistable loop—the dashed black line in Figure 2a. The observed spread in the FORC coercivity ($\Delta H_{c}=0.15$ Oe) is ascribed to the intrinsic fluctuations of the switching process in the single microwire—blue lines in Figure 2a. Due to the symmetry, this case can be compared with the one proposed by the Moving Preisach model when the moving parameter (a proportional factor to the interaction field) is zero [44]. Indeed, this symmetrical FORC distribution is a proper candidate to be modeled by the classical Preisach model, equivalent to a hysteron with $H_{c}=2.73$ Oe and $H_{u}=0$.

### 3.2. Sets of Two Bistable Microwires

**Figure 3:**
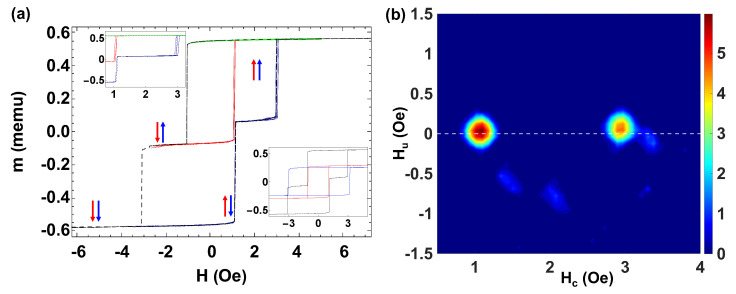
(**a**) FORC curves for two Fe${}_{77.5}$B${}_{15}$Si${}_{7.5}$ microwires with very different coercive fields. At the inset, the MHLs of each microwire are depicted (red and blue lines) as well as the loop (black line) when both microwires are placed together. (**b**) Corresponding FORC diagram.

To introduce the experimental observations in sets of bistable microwires, we start by considering the case of two bistable microwires with distinct individual coercive fields. The distance between the metallic inner cores is *d* = 12 μm. Figure 3a shows the FORC curves and the MHLs for a set of two coupled microwires with different coercive fields. The black dashed line denotes the MHL when both wires are placed together, characterized by two large Barkhausen jumps at $H_{sw,1}=1.12$ Oe and $H_{sw,2}=2.96$ Oe. The color arrows represent the position of the microwires in each part of the cycle. The MHLs of each individual microwire are depicted in the lower inset of Figure 3a. The red line corresponds to the microwire with $H_{0,1}=1.04$ Oe, and $m_{1}=0.271$ memu and the blue line to the microwire with $H_{0,2}=3.15$ Oe and $m_{2}=0.241$ memu.

Note that here, the interaction slightly increases/reduces the switching field of the microwire with smaller/larger individual coercivity. It is also worth mentioning that the switching fields, $H_{sw,i}$, in the MHL for this set of two microwires take relatively similar values as the coercive fields, $H_{0,i}$ of the individual microwires. This feature can be taken as a sign of the weak interaction field $H_{int}$ between the microwires as compared to the coercivity. Therefore, we can predict two FORC distributions located nearly at $H_{u}=0$.

In addition to the large Barkhausen jumps of the MHL, we can distinguish an intermediate plateau of width $\Delta H_{sw}=1.84$ Oe. In the present case, the magnetostatic interaction fields, calculated through Equation (8) are $H_{int,1}=0.82$ Oe and $H_{int,2}=0.92$ Oe. Along with the same considerations as for the case of two quasi-identical microwires, we find $\epsilon_{1}H_{int,2}=0.08$ Oe and $\epsilon_{2}H_{int,1}=-0.19$ Oe, and the respective the ϵ parameters are $\epsilon_{1}=0.10$ and $\epsilon_{2}=0.21$.

Figure 3b displays the FORC diagram for these two microwires showing two distributions at $\left(H_{c},H_{u}\right)=\left(1.10,0.04\right)$ Oe and $\left(H_{c},H_{u}\right)=\left(2.97,0.10\right)$ Oe. Note that, in this case, $H_{u,i}\approx \epsilon_{i}\left|H_{int,j}\right|$. The small displacement of the second distribution is due to the interaction of the first microwire. As the lower inset of Figure 3a shows, the second jump (dashed black line) occurs at $H_{sw}=2.93$ Oe, a smaller value than the individual microwire coercivity, $H_{0,2}=3.15$ Oe.

**Figure 4:**
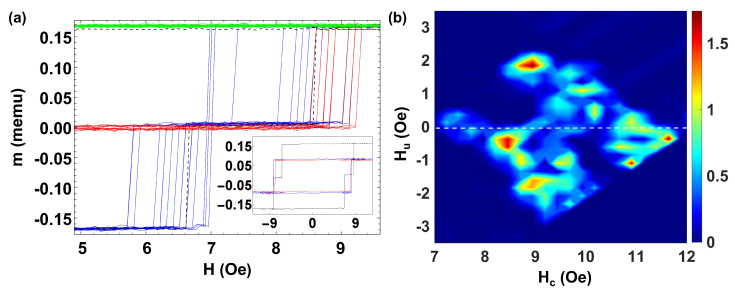
(**a**) FORC curves for two quasi-identical Fe${}_{77.5}$B${}_{15}$Si${}_{7.5}$ bistable amorphous microwires. The inset shows the MHLs for individual measurements: the red and blue lines correspond to the MHLs of the individual isolated microwires, while the dashed black line corresponds to the MHL when both wires are placed together. (**b**) Corresponding FORC diagram for the set of the two quasi-identical microwires.

Let us now analyze the case of two quasi-identical (nominally same length) pieces of magnetostrictive Fe${}_{77.5}$B${}_{15}$Si${}_{7.5}$ microwire. Figure 4 shows the FORC curves near the region of the switching process, the MHLs, and the FORC distribution for two quasi-identical microwires. A careful analysis of the experimental FORC curves is shown in Figure 4a. We observe a wide distribution of jumps produced by the experimental FORC curves with field steps of ΔH=ΔHr=0.1 Oe. The blue lines correspond to the first switch of one of the microwires, the red lines correspond to the second switch when just another microwire reverses, and the green lines correspond to the case in which none of the microwires reverses with a threshold value of $H_{r}=-6.5$ Oe. The inset shows the MHLs measured individually (red and blue lines) $H_{0,1}=7.94$ Oe and $H_{0,2}=8.11$ Oe, and magnetic moments $m_{1}=0.087$ memu and $m_{2}=0.082$ memu, respectively. The dashed black line corresponds to the MHL for both microwires placed side by side with their axes parallel to the applied field.

The small differences between the coercive fields and magnetic moments are ascribed to the slight difference in the total length of each microwire in the manual cutting procedure, a process that can also induce small modifications in the nucleation/depinning of the single domain wall resulting in small changes in the coercivity. The distance between the metallic cores is the sum of the glass-coating thicknesses, *d* = 15 μm. Note that although the glass-coated microwires are touching, the micrometric Pyrex coat avoids any magnetic exchange coupling between microwires.

The saturation magnetic moment is the addition of those for the individual microwires and two major Barkhausen jumps. The magnetization reversal in each microwire characterizes the overall loop shape. These jumps occur at $H_{sw,1}=6.60$ Oe and $H_{sw,2}=8.46$ Oe. Indeed, because of the magnetostatic interaction described by Equation (7), the first/second switching occurs at field $H_{sw,1}$/$H_{sw,2}$ smaller/larger than the coercive field of the individual microwire $H_{0,1}$/$H_{0,2}$. In addition, we observe an intermediate flat or plateau region that is determined, apart from the difference in coercivities of individual microwires, by magnetostatic interactions, $H_{int}$, between the microwires. The magnetostatic interaction field through Equation (8) is $H_{int,2}=0.22$ Oe and $H_{int,1}=0.24$ Oe. Considering Equation (7), we estimate that $\epsilon_{1}H_{int,2}=-1.34$ Oe and $\epsilon_{2}H_{int,1}=0.35$ Oe, so that the ϵ parameters are $\epsilon_{1}=6.02$ and $\epsilon_{2}=1.47$.

The corresponding FORC diagram is depicted in Figure 4b. Two symmetric distributions at $\left(H_{c},H_{u}\right)=\left(8.95,1.84\right)$ Oe, $\left(H_{c},H_{u}\right)=\left(8.97,-1.84\right)$ Oe appears at both sides of the $H_{c}$ axis. Note that these values of $H_{c}$ are slightly higher than those of $H_{0,1}$ while $H_{u}\approx \epsilon_{2}H_{int,1}$. A third distribution at $\left(H_{c},H_{u}\right)=\left(8.45,-0.37\right)$ can be related to the cases in which both microwires reverse at the same time. The two smaller peaks at $\left(H_{c},H_{u}\right)=\left(10.91,-1.10\right)$ Oe, and $\left(H_{c},H_{u}\right)=\left(11.64,-0.37\right)$ Oe together with the rest of the diffuse distributions do not seem to have a clear physical meaning. Indeed, we interpret these data by assuming that the FORC algorithm treats the intrinsic fluctuations of the switching process as interactions, which are reflected in the diagram as extra peaks and thus break the direct interpretation of the diagram. Furthermore, let us also remark that due to the calculation of the mixed second derivative in Equation (10), data points near *H* = $H_{sw}$ might produce slope discontinuities [45]. These calculation artifacts amplify measurement irregularities and give rise to noisier FORC diagrams [46,47].

### 3.3. Sets of Multiple Bistable Microwires

**Figure 5:**
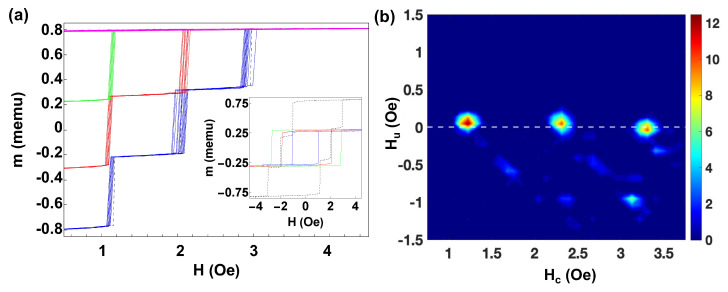
(**a**) FORC curves and individual MHLs (at the inset) for three Fe${}_{77.5}$B${}_{15}$Si${}_{7.5}$ microwires with different coercive fields. (**b**) Corresponding FORC diagram for this set of microwires.

For a deeper understanding, we performed measurements for sets of a higher number of microwires. Figure 5 shows the FORC curves and the MHLs for a set of three microwires with different individual coercivities. The FORC curves with different sequences of jumps marked in different colors are shown in Figure 5a. The inset represents the MHLs for the individual microwires, the blue line corresponds to a microwire with $H_{0,1}=1.04$ Oe and $m_{1}=0.271$ memu, the red line corresponds to a microwire with $H_{0,2}=1.99$ Oe and $m_{2}=0.301$ memu, while the green line corresponds to a microwire with $H_{0,3}=3.09$ Oe and $m_{2}=0.241$ memu. The black dashed line corresponds to the MHL when the three microwires are placed together. Three clear Barkhausen jumps at $H_{sw,1}=1.11$ Oe, $H_{sw,2}=1.99$ Oe, and $H_{sw,3}=2.94$ Oe and two plateaux of width $\Delta H_{sw,1}=0.84$ Oe and $\Delta H_{sw,2}=0.95$ Oe are distinguished. Although the interaction field between the microwires is weak, from the comparison between coercivities of isolated, $H_{0,i}$, and interacting microwires, $H_{sw,i}$, we deduce that the interaction is positive for the microwire with smaller isolated coercivity (e.g., $H_{int}$ reinforces $H_{0,1}$), negative for the microwire with higher isolated coercivity (e.g., $H_{int}$ reduces $H_{0,3}$) and vanishes for the intermediate one.

The corresponding FORC diagram is depicted in Figure 5b. We can distinguish three coercivity distributions along the $H_{c}$ axis. A slight displacement along the $H_{u}$ axis due to the magnetostatic interactions can be observed. In this case, for the microwire with $H_{0,1}=1.05$ Oe (blue line at the inset of Figure 5a), the corresponding FORC distribution is located at $\left(H_{c},H_{u}\right)=\left(1.22,0.04\right)$ Oe, for the microwire with $H_{0,2}=1.99$ Oe (red line at the inset of Figure 5a) the FORC distribution is at $\left(H_{c},H_{u}\right)=\left(2.31,0.04\right)$ Oe. Finally, the microwire with $H_{0,3}=3.09$ Oe (green line at the inset of Figure 5a) the FORC distribution is located at $\left(H_{c},H_{u}\right)=\left(3.31,-0.04\right)$ Oe. With the same considerations as with two microwires, we estimate the interaction fields as $\epsilon_{1}H_{int,j}=0.07$ Oe, $\epsilon_{2}H_{int,j}=0.0$ Oe, and $\epsilon_{3}H_{int,j}=-0.15$ Oe, which as in the previous cases are $\approx H_{u,i}$. Note also that $H_{sw,i}$ < $H_{c,i}$ for the three microwires.

**Figure 6:**
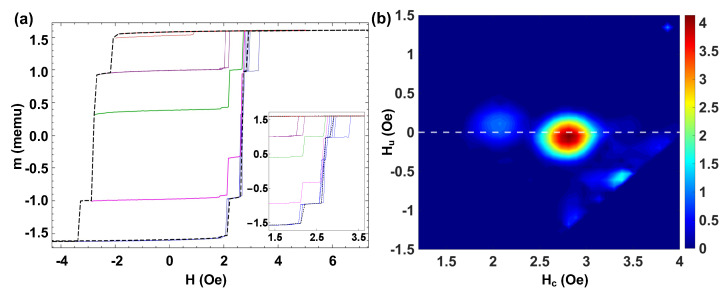
(**a**) MHL (black line) and FORC curves (color lines) for a set of five microwires with $H_{0}=\left\{2.69,2.74,2.86,3.08,3.29\right\}$ Oe. (**b**) Corresponding FORC diagram for this set of microwires.

To discuss a next step case on the transition from the discrete to the many interacting microwires case, we have extended the analysis to the study of a set of five bistable microwires placed side by side. Figure 6a shows the MHL (dashed black line) and the FORC curves (blue lines) for a set of five microwires with $H_{0,i}=\left\{2.69,2.74,2.86,3.08,3.29\right\}$ Oe and respective individual magnetic moments $m_{i}=\left\{0.306,0.314,0.317,0.302,0.288\right\}$ memu. The coercive fields of the individual microwires are distributed within a reduced range, and the plateaux are partly masked between the magnetization jumps. We can distinguish two plateaux of widths $\Delta H_{sw,1}=0.40$ Oe, $\Delta H_{sw,2}=0.11$ Oe.

Figure 6b shows the corresponding FORC distribution with a single FORC distribution with some significant broadness, which can be ascribed to such individual coercivities. A prominent peak at $\left(H_{c},H_{u}\right)=\left(3.04,-0.04\right)$ Oe is obtained, where $H_{c}$ is within the range of coercivities $H_{0,i}$ for individual microwires, and the interaction field $H_{u}$ is notably weak. We should note that the width of the FORC distribution along the $H_{c}$ axis is $\Delta H_{c}=0.61$ Oe, which correlates with the difference between the largest and smallest coercive field of the five microwires observed in the MHL. Interestingly, a fine observation of the individual magnetization jumps in the FORC curves of Figure 6a allows us to correlate them with more diffuse distributions in the FORC diagrams of Figure 6b. Particularly, the more diffuse distribution at $\left(H_{c},H_{u}\right)=\left(2.08,0.12\right)$ Oe can be ascribed to the microwire with the smallest coercive field. This is especially remarkable in the upper part of Figure 6a because of the interaction with the rest of the microwires, which are already in the “up” position, as this microwire reverses at a smaller switching field, $H_{sw}$, than its coercive field $H_{0}$.

**Figure 7:**
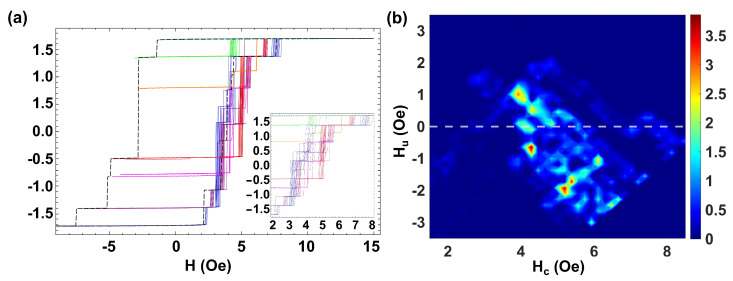
(**a**) MHL (black line) and FORC curves (color lines) for seven Fe${}_{77.5}$B${}_{15}$Si${}_{7.5}$ microwires with different coercive fields $H_{c}\in \left(2,8\right)$ and κ∈(0.25,0.4) placed side by side. (**b**) Corresponding FORC diagram.

Finally, to study the range of many interacting microwires, we discuss the case of a larger number of microwires with coercivity distributed in a range of different values (e.g., including a few microwires with similar coercivities $H_{0,i}\in \left(2.5,8.7\right)$ Oe, and some others with well different values) as well as individual magnetic moments $m_{i}\in \left(0.14,0.47\right)$ memu. Figure 7a shows the MHL (dashed black line) and the FORC curves (color lines) for this set of seven microwires.

We should remark on its clear asymmetric shape that indicates a different sequence of microwires reversing magnetization (e.g., when magnetizing positively and negatively) correlated to both their magnetostatic interactions and the switching field fluctuations of the individual microwires. Indeed, when applying the largest positive or negative magnetic fields, the configuration at the end of the microwires is not always the same. Thus, slight differences in the magnetic configuration in the near-saturated state determine the depinning and propagation of the domain wall. As an example, for the single MHL (black dashed line), when magnetizing towards positive values, the two microwires with the lowest coercive field reverse at the same time at $H_{sw}$ = 2.1 Oe, although when magnetizing towards negative values, one reverses at $H_{sw}$ = −1.4 Oe, the other switches at $H_{sw}$ = −2.8 Oe together with another two microwires. On the other hand, it should be noted that when we perform the FORC curves, the field step towards positive fields is quite small ΔH = 0.05, while the return in the opposite direction to $H_{r}$ from saturation is done in a single step. This difference also implies the presence of asymmetry in the cycles.

A descriptive video of this process is supplied in the Supplementary Information. A quite rich spectrum of Barkhausen jumps in the FORC curves depicted in blue color is found. Here, the intrinsic coercivity fluctuations pointed out in the case of a single microwire become quite relevant. Those fluctuations make some microwires reverse magnetization at smaller/larger fields modifying the resulting magnetostatic interactions. Indeed, each of the minor FORC curves has a different distribution of jumps at different switching fields, $H_{sw,i}$. Since the microwires are placed side-by-side in a planar configuration, the introduction of an increasing number of microwires modifies the interaction between the closer microwires. $H_{int}$ is ascribed to the distribution of magnetic charges along the microwires. In the case of isolated microwires, these charges are essentially located in the neighborhood of the ends; thus, they can be taken as nearly ideal magnetic dipoles, the polarity of which reverses upon magnetization reversal in each microwire. However, when approaching the microwires in a planar configuration, the magnetic charges redistribute along the microwires so the effective interaction fields, $H_{int}$, become complex to quantify and cannot be precisely correlated to the $H_{u}$ position of the FORC distribution.

Figure 7b shows the corresponding FORC diagram, where we identify a rich distribution in coercivities and interaction fields. We observe six dominant peaks at $\left(H_{c},H_{u}\right)=\left(3.92,1.00\right)$ Oe, $\left(H_{c},H_{u}\right)=\left(4.28,0.46\right)$ Oe, $\left(H_{c},H_{u}\right)=\left(4.28,-0.64\right)$ Oe, $\left(H_{c},H_{u}\right)=\left(5.20,-1.91\right)$ Oe, $\left(H_{c},H_{u}\right)=\left(5.38,-1.73\right)$ Oe, and $\left(H_{c},H_{u}\right)=\left(6.11,-1.00\right)$ Oe and an elongated distribution along the Hc axis at $\left(H_{c},H_{u}\right)=\left(4.2,0.0\right)$ Oe that can be related to the microwires with similar coercive fields. Two more diffused distributions at $\left(H_{c},H_{u}\right)=\left(6.48,-2.46\right)$ Oe and $\left(H_{c},H_{u}\right)=\left(6.66,-2.28\right)$ Oe are correlated to the microwires with higher coercive fields. Such a rich distribution is due to the intrinsic fluctuations of coercivity of the individual microwires that can be of the same order as the difference of coercivity of isolated microwires. In addition, several green-blue distributions in the range $H_{c}=\left(3.92,7.34\right)$ Oe are also observed. The presence of these additional distributions with no physical meaning makes the interpretation of the FORC diagram complex, particularly the identification of the FORC distributions related to the interactions between individual microwires, which cannot be directly related to the corresponding asymmetric MHL.

## 4. Conclusions

The versatility of the FORC method to describe the reversal behavior for single and several sets of coupled glass-coated amorphous microwires with Fe${}_{77.5}$B${}_{15}$Si${}_{7.5}$ composition showing individual bistable magnetic character has been tested. The MHL is characterized by plateaux whose widths are related to both the magnetostatic interactions between them and the differences in their individual coercive fields. Such interactions are modified “discontinuously” as the individual microwires reverse magnetization and introduce fluctuations in the switching field value, $H_{sw}$.

FORC analysis allows the determination of the coercive field distributions in many-body systems as well as their interaction fields. The present case is a non-standard one since the magnetization switching of coupled microwires with bistable behavior proceeds a giant Barkhausen jump at a field that presents intrinsic fluctuations. The main characteristics of the FORC distribution for positive magnetostriction bistable microwires are: (i) a single peak along the $H_{c}$ axis related to the individual coercive fields for the non-interacting cases, (ii) a discrete number of peaks related to the isolated microwire coercivity displaced from the $H_{c}$ axis due to the magnetostatic interaction, and (iii) the presence of extra peaks due to both their magnetostatic interactions and the switching field fluctuations of the individual microwires.

The different kinds of sets of bistable microwires point out the complexity of analyzing and interpreting the obtained FORC diagrams. The FORC analysis for sets of microwires with different coercivity (e.g., cases of two, three, and five microwires) concludes with a positive determination of coercivity distributions around the single coercivity values. On the other hand, in the cases with similar coercivity (two quasi-identical and seven microwires), a rich distribution of jumps at different $H_{sw}$ values is observed as a consequence of both the magnetostatic interactions and the intrinsic fluctuations of the $H_{sw}$. These intrinsic fluctuations are reflected in the diagram as additional peaks. Hence, in those cases, the most prominent peaks are connected with the individual coercive fields of the microwires, while the additional diffuse distributions with no physical meaning break the exact interpretation of the FORC diagrams. Furthermore, data points near *H* = $H_{sw}$ might produce calculation artifacts that give rise to noisier FORC diagrams with extra peaks.

As an example of an intermediate case to the standard many-body problem, the case of seven different microwires shows that the most complex behavior with a FORC diagram with a rich structure of coercivity distributions and interaction fields is due to the intrinsic fluctuations of coercivity of the individual microwires that can be of the same order as the difference of coercivity of isolated microwires. This feature modifies the sequence of reversal processes in the multiple FORC curves reflected in the FORC diagram as additional diffuse distributions that cannot be directly related to the asymmetric MHL.

To summarize, we conclude that for the less or non-interacting cases, the physical interpretation of the FORC distribution’s spread of the single microwires is related to the switching field fluctuations of the individual microwires. In turn, in the cases where magnetostatic interactions are significant, the MHLs become asymmetric due to fluctuations in the depinning field for the propagation of the domain walls. The appearance of additional distributions on the FORC diagram gives more information than standard MHLs regarding the intrinsic fluctuations of the switching process and the magnetostatic interactions. Since many of the applications of amorphous microwires are based on fast magnetization switching and domain wall propagation, the present study points out the complexity of the mechanisms of magnetic interactions in sets of microwires. Moreover, it represents the first investigation of the role of such interactions on sets of bistable elements as intermediate to the standard many-body systems.

## Data Availability

Not applicable.

## Supplementary Materials

The following supporting information can be downloaded at: https://www.mdpi.com/article/10.3390/ma16062131/s1, Video S1: FORC curves sequence for the seven microwires.

## Abbreviations

The following abbreviations are used in this manuscript:

FORC	first-order reversal curves
MHL	major hysteresis loop
VSM	vibrating sample magnetometer
$H_{c}$	coercive field
$H_{u}$	interaction field
$H_{r}$	reversal field
$H_{sw}$	switching field
$H_{0}$	coercive field of the isolated microwire
$H_{max}$	maximum applied field in FORC diagrams

## References

[1] Alam J., Bran C., Chiriac H., Lupu N., Óvári T.A., Panina L.V., Rodionova V., Varga R., Vázquez M., Zhukov A. (2020). Cylindrical micro and nanowires: Fabrication, properties and applications. J. Magn. Magn. Mater..

[2] Vázquez M. (2020). Magnetic Nano- and Microwires: Design, Synthesis, Properties and Applications.

[3] Larin V.S., Torcunov A.V., Zhukov A., González J., Vázquez M., Panina L. (2002). Preparation and properties of glass-coated microwires. J. Magn. Magn. Mater..

[4] Chiriac H., Óvári T.A. (1997). Amorphous glass-covered magnetic wires: Preparation, properties, applications. Prog. Mater. Sci..

[5] Vázquez M., Zhukov A.P. (1996). Magnetic properties of glass-coated amorphous and nanocrystalline microwires. J. Magn. Magn. Mat..

[6] Zhukov A. (2001). Domain wall propagation in a Fe-rich glass-coated amorphous microwire. Appl. Phys. Lett..

[7] Zhukov A., Ipatov M., Talaat A., Blanco J.M., Hernando B., Gonzalez-Legarreta L., Suñol J.J., Zhukova V. (2017). Correlation of Crystalline Structure with Magnetic and Transport Properties of Glass-Coated Microwires. Crystals.

[8] Vázquez M., Chiriac H., Zhukov A., Panina L., Uchiyama T. (2011). On the state-of-the-art in magnetic microwires and expected trends for scientific and technological studies. Phys. Status Solidi A.

[9] Corte-Leon P., Zhukova V., Chizhik A., Blanco J.M., Ipatov M., Gonzalez-Legarreta L., Zhukov A. (2020). Magnetic Microwires with Unique Combination of Magnetic Properties Suitable for Various Magnetic Sensor Applications. Sensors.

[10] Qin F.-X., Peng H.-X. (2013). Ferromagnetic microwires enabled multifunctional composite material. Prog. Mater. Sci..

[11] Sampaio L.C., Sinnecker E.H.C.P., Cernicchiaro G.R.C., Knobel M., Vázquez M., Velázquez J. (2000). Magnetic microwires as macrospins in a long-range dipole-dipole interaction. Phys. Rev. B.

[12] Pereira A., Denardin J.C., Escrig J. (2009). How do magnetic mi-crowires interact magnetostatically?. J. Appl. Phys..

[13] Velázquez J., Vázquez M. (2002). An analysis of interacting bistable magnetic microwires: From ordered to chaotic behaviours. Physical B.

[14] Knobel M., Sampaio L.C., Sinnecker E.H.C.P., Vargas P., Altbir D. (2002). Dipolar magnetic interactions among magnetic microwires. J. Magn. Magn. Mater..

[15] Piccin R., Laroze D., Vargasand M.K.P., Vázquez M. (2007). Magnetostatic interactions between two magnetic wires. EPL.

[16] Pike R., Roberts P., Verosub K.L. (1999). Characterizing interactions in fine magnetic particle systems using first order reversal curves. J. App. Phys..

[17] Béron F., Clime L., Ciureanu M., Menard D., Cochrane R.W., Yelon A. (2006). First-order reversal curves diagrams of ferromagnetic soft nanowire arrays. IEEE Trans. Magn..

[18] Béron F., Carignan L.-P., Menard D., Yelon A., Lupu N. (2010). Extracting individual properties from global behaviour: First-order reversal curve method applied to magnetic nanowire arrays. Electrodeposited Nanowires and Their Applications.

[19] Dobrota C., Stancu A. (2013). What does a first-order reversal curve diagram really mean? A study case: Array of ferromagnetic nanowires. J. Appl. Phys..

[20] Rotaru A., Lim J.H., Lenormand D., Diaconu A., Wiley J.B., Postolache P., Stancu A., Spinu L. (2011). Interactions and reversal-field memory in complex magnetic nanowire arrays. Phys. Rev. B.

[21] Stancu A., Pike C., Stoleriu L., Postolache P., Cimpoesu D. (2003). Micromagnetic and Preisach analysis of the First Order Reversal Curves (FORC) diagram. J. Appl. Phys..

[22] Kolesnikova V., Martínez-García J.C., Rodionova V., Rivas M. (2020). Study of bistable behaviour in interacting Fe-based microwires by first order reversal curves. J. Magn. Magn. Mater..

[23] Jiang L., Yang C., Song Z., Takemura Y. (2022). Magnetic Structure of Wiegand Wire Analyzed by First-Order Reversal Curves. Materials.

[24] Rivas M., Martínez-García J.C., Škorvánek I., Marcin J., Svec P., Gorria P. (2015). Magnetostatic interaction in soft magnetic bilayer ribbons unambiguously identified by first-order reversal curve analysis. Appl. Phys. Lett..

[25] Belyaev V.K., Murzin D., Martínez-García J.C., Rivas M., Andreev N.V., Kozlov A.G., Samardak A.Y., Ognev A.V., Samardak A.S., Rodionova V. (2021). FORC-Diagram Analysis for a Step-like Magnetization Reversal in Nanopatterned Stripe Array. Materials.

[26] Béron F., Menard D., Yelon A. (2008). First-order reversal curve diagrams of magnetic entities with mean interaction filed: A physical analysis perspective. J. App. Phys..

[27] Torre E.D. (1966). Effect of Interaction on the Magnetization of Single Domain Particles. IEEE Trans. Audio Electroacoust..

[28] Rivas M., Gorria P., Munoz-Gomez C., Martínez-García J.C. (2017). Quasi-Static AC FORC Measurements for Soft Magnetic Materials and Their Differential Interpretation. IEEE Trans. Magn..

[29] Martínez-García J.C., Rivas M., Lago-Cachón D., García J.A. (2014). FORC differential dissection of soft biphase magnetic ribbons. J. Alloys Compd..

[30] Mayergoyz I. (1986). Mathematical models of hysteresis. IEEE Trans. Magn..

[31] Preisach F. (1935). Uber die magnetische. Nachwikung Z. Phys..

[32] Velázquez J., Pirota K.R., Vázquez M. (2003). About the Dipolar Approach in magnetostatically coupled bistable magnetic micro and nanowires. IEEE Trans. Magn..

[33] Aharoni A. (1996). Introduction to the Theory of Ferromagnetism.

[34] Jackson D. (1962). Classical Electrodynamics.

[35] Laroze D., Escrig J., Landeros P., Altbir D., Vazquez M., Vargas P. (2007). A detailed analysis of dipolar interactions in arrays of bi-stable magnetic nanowires. Nanotechnology.

[36] Chen D.X., Gómez-Polo C., Vázquez M. (1993). Magnetization profile determination in amorphous wires. J. Magn. Magn. Mater..

[37] Gilbert D.A., Zimanyi G.T., Dumas R.K., Winklhofer M., Gomez A., Eibagi N., Vicent J.L., Liu K. (2014). Quantitative decoding of interactions in tunable nanomagnet arrays using first order reversal curves. Sci. Rep..

[38] Clime L., Stancu A., Ciureanu P., Yelon A. (2004). First order reveral curves diagram deduced by a Shepard method for bivariate interpolation of scattered data. J. Optoelectron. Adv. Mater..

[39] Pike R. (2003). Fist-order reversal curve diagrams and reversible magnetization. Phys. Rev. B.

[40] Davies J.E., Hellwig O., Fullerton E.E., Denbeaux G., Kortright J.B., Liu K. (2004). Magnetization reversal of CoPt multilayers: Microscopic origin of high field magnetic irreversibility. Phys. Rev. B.

[41] Groß F., Ilse S.E., Schütz G., Gräfe J., Goering E. (2019). Interpreting first-order reversal curves beyond the Preisach model: An experimental permalloy microarray investigation. Phys. Rev. B.

[42] Samanifar S., Kashi M.A., Ramazani A., Alikhani M. (2015). Reversal modes in FeCoNi nanowire arrays: Correlation between magnetostatic interactions and nanowires length. J. Magn. Magn. Mater..

[43] Sergelius P., Fernandez J.G., Martens S., Zocher M., Böhnert T., Martinez V.V., Prida V.M.d., Görlitz D., Nielsch K. (2016). Statistical magnetometry on isolated NiCo nanowires and nanowire arrays: A comparative study. J. Phys. D Appl. Phys..

[44] Alejos O., Della Torre E. (2001). Magnetic aftereffect dependence on the moving parameter of the Preisach model. Phys. B Condens. Matter.

[45] Béron F., Clime L., Ciureanu M., Menard D., Cochrane R., Yelon A. (2007). Reversible and quasireversible information in first-order reversal curve diagrams. J. Appl. Phys..

[46] Béron F., Carignan L.P., Menard D., Yelon A. (2008). Magnetic Behavior of Ni/Cu Multilayer Nanowire Arrays Studied by First-Order Reversal Curve Diagrams. IEEE Trans. Magn..

[47] Béron F., Clime L., Ciureanu M., Ménard D., Cochrane R.W., Yelon A. (2008). Magnetostatic Interactions and Coercivities of Ferromagnetic Soft Nanowires in Uniform Length Arrays. J. Nanosci. Nanotechnol..

